# A Study to Derive Equivalent Mechanical Properties of Porous Materials with Orthotropic Elasticity

**DOI:** 10.3390/ma14185132

**Published:** 2021-09-07

**Authors:** Changmin Pyo, Younghyun Kim, Jaewoong Kim, Sungwook Kang

**Affiliations:** 1 Smart Mobility Materials and Components R&D Group, Korea Institute of Industrial Technology, Gwangju 61012, Korea; changmin@kitech.re.kr (C.P.); kyh1927@kitech.re.kr (Y.K.); 2 Precision Mechanical Process and Control R&D Group, Korea Institute of Industrial Technology, Jinju 52845, Korea; swkang@kitech.re.kr

**Keywords:** equivalent mechanical properties, porosity, finite element method, orthotropic elasticity

## Abstract

The need for diverse materials has emerged as industry becomes more developed, and there is a need for materials with pores in various industries, including the energy storage field. However, there is difficulty in product design and development using the finite element method because the mechanical properties of a porous material are different from those of a base material due to the pores. Therefore, in this study, a Python program that can estimate the equivalent property of a material with pores was developed and its matching was verified through comparison with the measurement results. For high-efficiency calculation, the pores were assumed to be circular or elliptical, and they were also assumed to be equally distributed in each direction. The material with pores was assumed to be an orthotropic material, and its equivalent mechanical properties were calculated using the equivalent strain and equivalent stress by using the appropriate material property matrix. The material properties of a specimen with the simulated pores were measured using UTM, and the results were compared with the simulation results to confirm that the degree of matching achieved 6.4%. It is expected that this study will contribute to the design and development of a product in the industrial field.

## 1. Introduction

When developing a new product, the materials constituting the product must have target mechanical and electrical properties under its operating conditions. In the past, a product suitable for a specific situation was manufactured by using a single material, but it was difficult to satisfy complex design conditions. Recently, various materials including composite materials have been used to satisfy complex design conditions, contributing to the development of products and technologies.

In addition, the demand for porous materials is steadily increasing; specifically, the need for porous materials is rising in fields of chemical engineering such as pharmaceuticals and catalysts, as well as in the field of energy storage. Related research has also been ongoing [1,2,3,4].

Moreover, many materials such as bones, teeth, and other biomaterials in medicine and dentistry industries are porous materials [5], and porous tantalum for dental implantology has porosities [6].

Concept design–basic design–detail design–mockup production and verification procedures are essential to develop products and technologies. To minimize trial and error, it is advantageous to verify design feasibility through the finite element method at the design stage, and this is an approach widely used in academia as well as in industry. The finite element method has the advantage of being able to verify design feasibility at a much lower cost compared with experiments or deriving the optimal design through case studies under various conditions [7,8].

However, when the finite element method is used for a material with pores, the pores show properties that differ from the mechanical properties of an existing base material. This may distort the results of the finite element method, and is a limitation when using the finite element method for the materials. To solve this problem, a method is used to estimate and utilize equivalent mechanical properties while simplifying modeling by applying the homogenization method for composite materials [9,10,11,12].

However, there has been little research on the application of the homogenization method to materials with pores. Therefore, this study addressed how to calculate equivalent mechanical properties that reflect the pores, for a material that has pores. In prior studies, a simulation technique that can derive isotropic material properties under the plane stress condition, which is a material property that can be used in a 2D shell, was developed and was verified under the two-dimensional orthotropic elasticity in plane stress that is symmetrical in the in-plane direction [13,14]. In this study, the elastic modulus and Poisson’s ratio, which are the main equivalent properties of porous materials, were derived under the condition of three-dimensional orthotropic elasticity. Assuming that the shape of pores is symmetrical in the in-plane direction, and that the pores are uniformly distributed in the in-plane direction, a unit cell was defined and equivalent properties were calculated. Thus, the elastic modulus and Poisson’s ratio were measured through a universal test machine (UTM) by preparing a material specimen with the same simulation conditions. As a result, the similarity between the value obtained through the simulation and the value measured through the experiment was observed, so the validity of this simulation was verified.

## 2. Prior Research and Background Theory

### 2.1. Study on Equivalent Properties Derivation

The homogenization method was mainly used to derive the equivalent physical properties. Homogenization methods include the representative unit cell method [15] and the representative equivalent continuum method [16], but the most dominant homogenization method is the representative volume element (RVE) method [9,10,11,12]. The representative volume element method is applied to estimate the stiffness of fiber composites [9] or to estimate the equivalent properties of reinforced plastics mixed with multidirectional fibers [10]. It has also been used in studies to estimate the equivalent properties of an anisotropic material [11,12].

**Figure 1:**
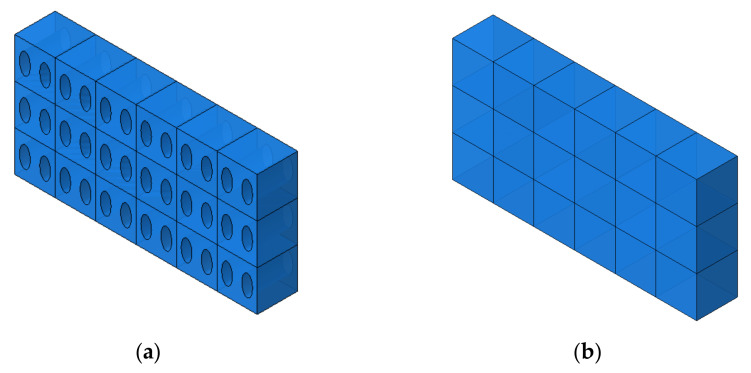
(**a**) Shape of unit cell before homogenization. (**b**) Shape of unit cell after homogenization.

The representative volume element assumes that the strain energy before and after homogenization should be the same. When various materials or pores are distributed within a unit volume, it is assumed that all material properties within a unit volume are the same (Figure 1).

The related governing equations are shown as Equations (1)–(5) below [9,10,11,12]. Equation (1) means that the strain energy before and after homogenization is the same. Equation (2) means that the strain energy before homogenization has a relationship with the sum of the strain energies for each element in the entire unit volume. Equation (3) indicates that the energy after homogenization is related to the average strain energy, which is obtained by multiplying the average value of the stress for each element and the average value of the strain for each element, multiplied by the volume. Equation (4) is a method to calculate the average stress, and Equation (5) is a method to calculate the average strain.

(1) $$U^{*}=U$$

(2) $$U^{*}=\frac{1}{2}\int_{V_{RVE}}\sigma_{ij}\epsilon_{ij}dV$$

(3) $$U=\frac{1}{2}\bar{\sigma_{ij}}\bar{\epsilon_{ij}}V_{RVE}$$

(4) $$\bar{\sigma_{ij}}=\frac{1}{V_{RVE}}\int_{V^{*}}\sigma_{ij}\left(x,y,z\right)dV$$

(5) $$\bar{\varepsilon_{ij}}=\frac{1}{V_{RVE}}\int_{V^{*}}\varepsilon_{ij}\left(x,y,z\right)dV$$

U*: strain energy before homogenization; *U*: strain energy after homogenization; V*: total volume before homogenization; $V_{RVE}$: volume after homogenization; $\sigma_{ij}\left(x,y,z\right)$: stress per element; $\varepsilon_{ij}\left(x,y,z\right)$: strain per element; $\bar{\sigma_{ij}}$: stress weighted average for each element; $\bar{\varepsilon_{ij}}$: strain weighted average for each element.

### 2.2. Orthotropic Elasticity

**Figure 2:**
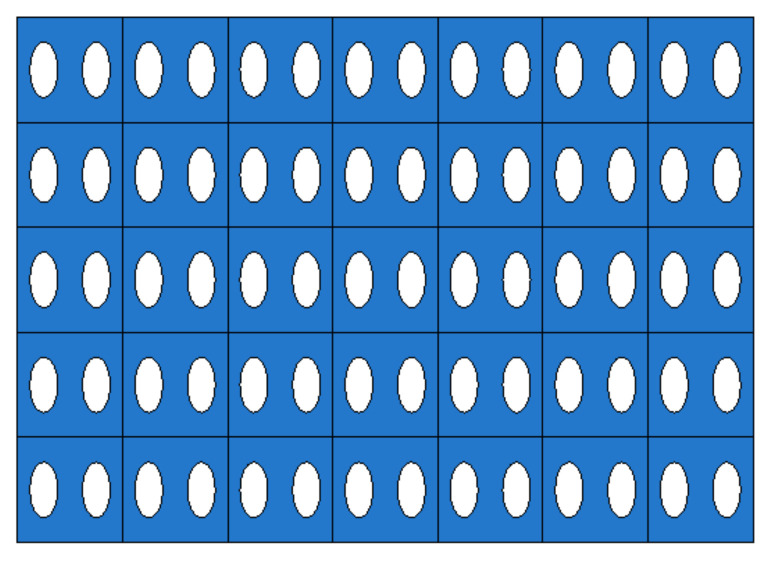
Shape of orthotropic elasticity.

In this study, it was assumed that the pores are asymmetric in the in-plane direction, and that the distribution of the pores is uniform in each direction (Figure 2). The size of pores is also irregular, and the distribution is not uniform, so it is correct to think that a material with pores has anisotropic properties. In this case, all materials should be verified through experiments and measurements. If a material is too thin or is toxic, it is difficult to measure it using UTM, etc., and it is not suitable for the purpose of performing simulation using FEM. Therefore, in this study, a material with pores was assumed to be an orthotropic material, which is thought to be a more suitable property for use in the industrial field.

The elastic modulus determinant of a perfectly anisotropic material is shown in Equation (6) [17]. In addition, the elastic modulus determinant of an orthotropic material is shown in Equation (7) [17]. Because the shape of pores is difficult to standardize and their distribution is not uniform, it is more realistic to assume that the material is a perfectly anisotropic material. As this study estimates the equivalent properties of a material by approximating the shape and distribution of pores, the material was assumed to be an orthotropic material, and the elastic modulus determinant was applied accordingly.


(6) $$\left[\begin{array}{c} \bar{\sigma_{x}}\\ \bar{\sigma_{y}}\\ \bar{\sigma_{x}}\\ \bar{\sigma_{xy}}\\ \bar{\sigma_{xz}}\\ \bar{\sigma_{yz}} \end{array}\right]=\left[\begin{array}{cc} \begin{array}{ccc} D_{1111} & D_{1122} & D_{1133}\\ & D_{2222} & D_{2233}\\ & & D_{3333} \end{array} & \begin{array}{ccc} D_{1112} & D_{1113} & D_{1123}\\ D_{2212} & D_{2213} & D_{2223}\\ D_{3312} & D_{3313} & D_{3323} \end{array}\\ \begin{array}{ccc} Sym. & & \end{array} & \begin{array}{ccc} D_{1212} & D_{1213} & D_{1223}\\ & D_{1313} & D_{1323}\\ & & D_{2323} \end{array} \end{array}\right]\left[\begin{array}{c} \bar{\epsilon_{x}}\\ \bar{\epsilon_{y}}\\ \bar{\epsilon_{z}}\\ \bar{\epsilon_{xy}}\\ \bar{\epsilon_{xz}}\\ \bar{\epsilon_{yz}} \end{array}\right]$$

(7) $$\left[\begin{array}{c} {𝜀}_{11}\\ {𝜀}_{22}\\ {𝜀}_{33}\\ {𝛾}_{12}\\ {𝛾}_{13}\\ \tau_{23} \end{array}\right]=\left[\begin{array}{c} \begin{array}{cccccc} 1/E_{1} & -\nu_{21}/E_{2} & -\nu_{31}/E_{3} & 0 & 0 & 0\\ -\nu_{12}/E_{1} & 1/E_{2} & -\nu_{32}/E_{3} & 0 & 0 & 0\\ -\nu_{13}/E_{1} & -\sigma_{23}/E_{2} & 1/E_{3} & 0 & 0 & 0\\ 0 & 0 & 0 & 1/G_{12} & 0 & 0\\ 0 & 0 & 0 & 0 & 1/G_{13} & 0\\ 0 & 0 & 0 & 0 & 0 & 1/G_{23} \end{array} \end{array}\right]\left[\begin{array}{c} \sigma_{11}\\ \sigma_{22}\\ \sigma_{33}\\ \tau_{12}\\ \tau_{13}\\ \tau_{23} \end{array}\right]$$

For reference, the elastic modulus determinant of an isotropic material under a plane stress condition is shown in Equation (8) [16], and the elastic modulus determinant of an orthotropic material under a plane stress condition is shown in Equation (9).

(8) $$\left[\begin{array}{c} \bar{\epsilon_{x}}\\ \bar{\epsilon_{y}}\\ \bar{\gamma_{xy}} \end{array}\right]=\left[\begin{array}{ccc} \frac{1}{E_{x}} & -\frac{\bar{\nu_{yx}}}{\bar{E_{y}}} & 0\\ -\frac{\bar{\nu_{xy}}}{\bar{E_{x}}} & \frac{1}{\bar{E_{y}}} & 0\\ 0 & 0 & \frac{1}{\bar{G_{xy}}} \end{array}\right]\left[\begin{array}{c} \bar{\sigma_{x}}\\ \bar{\sigma_{y}}\\ \bar{\tau_{xy}} \end{array}\right]$$

(9) $$\left[\begin{array}{c} \bar{\epsilon_{x}}\\ \bar{\epsilon_{y}}\\ \bar{\gamma_{xy}} \end{array}\right]=\frac{1}{\bar{E}}\left[\begin{array}{ccc} 1 & -\bar{\nu } & 0\\ -\bar{\nu } & 1 & 0\\ 0 & 0 & 2\left(1+\bar{\nu }\right) \end{array}\right]\left[\begin{array}{c} \bar{\sigma_{x}}\\ \bar{\sigma_{y}}\\ \bar{\tau_{xy}} \end{array}\right]$$

## 3. Deriving Equivalent Properties Using Simulation

### 3.1. Computer Software

In this study, Abaqus 2020 by Dassault Systèmes was used as a finite element method program. Abaqus has been widely used in academia and industry due to its technical feasibility [18,19]. Using the result file derived from the finite element method program, the elastic modulus matrix was derived, and Python (Version 3.8.5, Python Software Foundation Beaverton, OR, USA) was applied to derive the equivalent properties of a material with pores from the elastic modulus matrix. Python has been widely used in research for data processing and matrix calculation because its validity has been verified [20].

### 3.2. Equivalence Derivation Process

**Figure 3:**
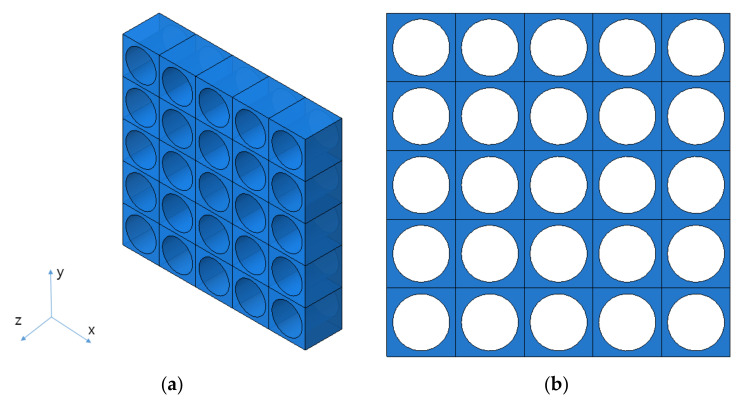
The shape of circular pores. (**a**) Isotropic view. (**b**) Top view.

**Figure 4:**
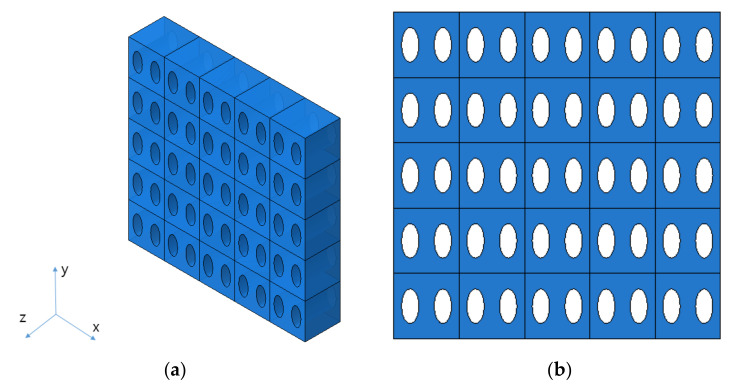
The shape of elliptical pores. (**a**) Isotropic view. (**b**) Top view.

In this study, it was assumed that the size and spacing of pores were uniform. The material properties were derived by using pores of two shapes, and they were compared through experiments. The shape of circular pores is shown in Figure 3, while the shape of elliptical pores is shown in Figure 4. Modeling was performed for the shape of a specimen to be used in the measurement, and verification was carried out as in Section 4. Circular and elliptical shapes were selected for ease of processing of a material specimen.

As shown in Figure 3 and Figure 4, unit cells were modeled and a constant displacement was applied as a load condition for each direction, and stress values were derived for each direction when the condition was applied.

To derive the equivalent stress, a weighted average was applied to the stress value of a mesh according to the volume size. In addition to the tensile stress in the in-plane X and Y directions and the shear stress in the XY direction, the tensile stress in the Z direction and the shear stress in the XZ and YZ directions were derived, respectively.

The elastic modulus and Poisson’s ratio of a representative volume element were calculated using the determinant obtained in Section 2.2 based on the equivalent strain in each direction, reflected as the load condition and the calculated equivalent stress in each direction.

### 3.3. Determination of Unit Cell Model and Its Shape

**Figure 5:**
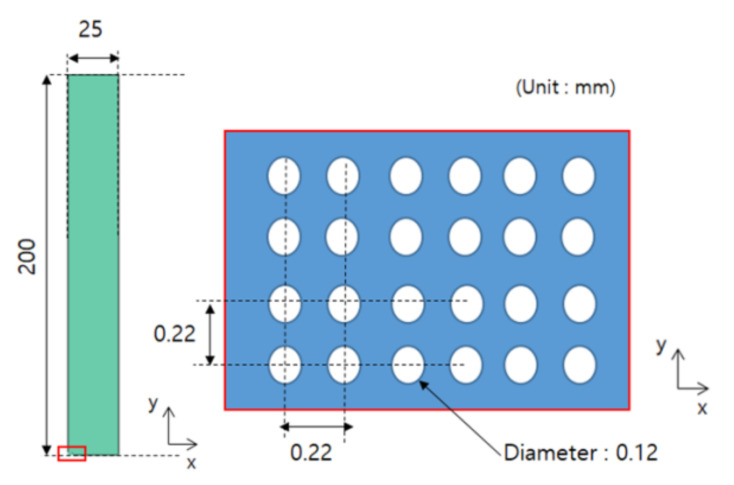
Dimension and distribution of circular pores (Reprinted from Ref. [13]).

To verify the process in Section 3.2, a unit cell model that can be actually manufactured was selected. Through discussion with the specimen manufacturer, a model with a large number of pores in the smallest size was selected. The diameter of the circular pores was 120 μm, and the spacing between pores was 220 μm. The specimen was manufactured in a size of 25 mm in the X direction and 200 mm in the Y direction. For the comparison with the measurement results from UTM, they were manufactured in a size applicable to UTM. As a result, 113 pores in the X direction and 910 pores in the Y direction were obtained, and the shape of the designed specimen and that of the unit cell are shown in Figure 5. Actually, those tests were performed former studies [13,14], we used the test results from those studies.

Pattern processing was performed by applying an etching technique for pore processing, and a thickness of 30 μm was selected through discussion with the manufacturer for convenience and quality of production.

**Figure 6:**
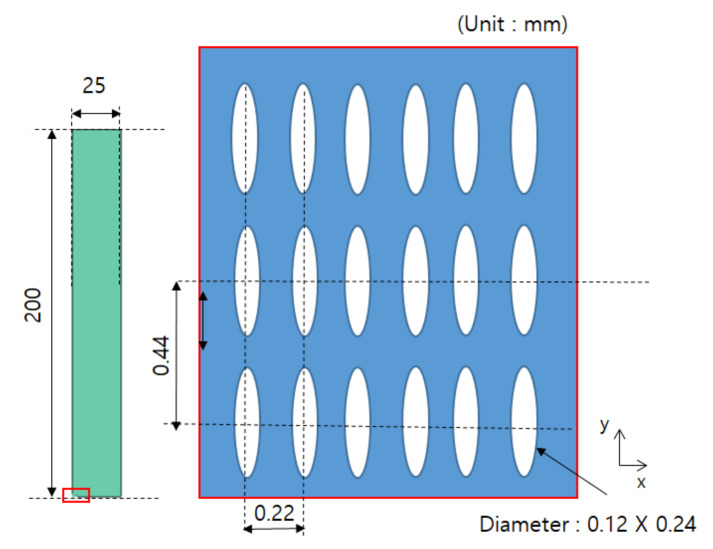
Dimension and distribution of elliptical pores (Reprinted from Ref. [14]).

**Figure 7:**
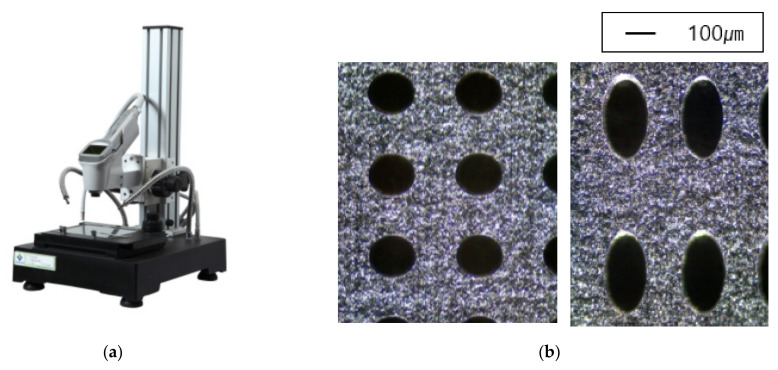
(**a**) Shape of optical microscope. (**b**) Image with optical microscope.

**Figure 8:**
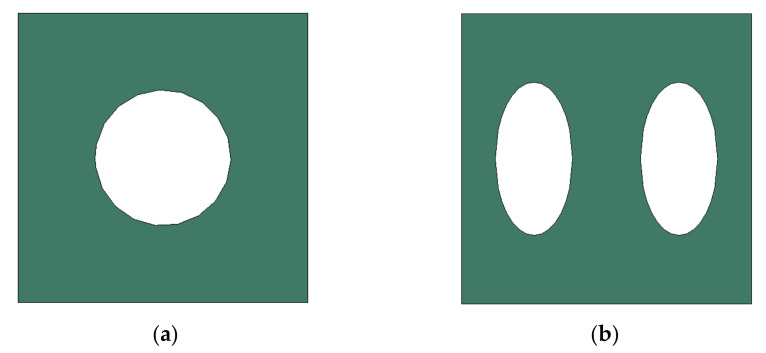
The shape of unit cell. (**a**) Circular pore. (**b**) Elliptical pore.

In addition to the circular pores, verification of the elliptical pores was also performed. The size and distribution of elliptical pores were determined through discussion with the manufacturer. The diameter was set to 120 μm × 240 μm, and the spacing between pores was determined to be 220 μm in the X direction and 440 μm in the Y direction. The size of a specimen was determined to be the same as that of a circular pore specimen, 25 mm × 200 mm × 30 μm, and the shape is shown in Figure 6. However the real dimensions were different from the design data; thus, we checked real dimensions such as the hole diameter. The pattern processing shape of a processed specimen was checked using an optical scope. Its size and shape were measured by applying the optical microscope (i-megascope System2 Mega pixels, Sometech, Seoul, Korea). The shape of an optical microscope is shown in Figure 7. Absolutely, we applied the real dimensions for the simulation. The information is in Table 1. Each unit cell for simulation is defined in Figure 8. The base material of each specimen was SUS304, and its mechanical properties are shown in Table 2.

### 3.4. Simulation Procedure

**Figure 9:**
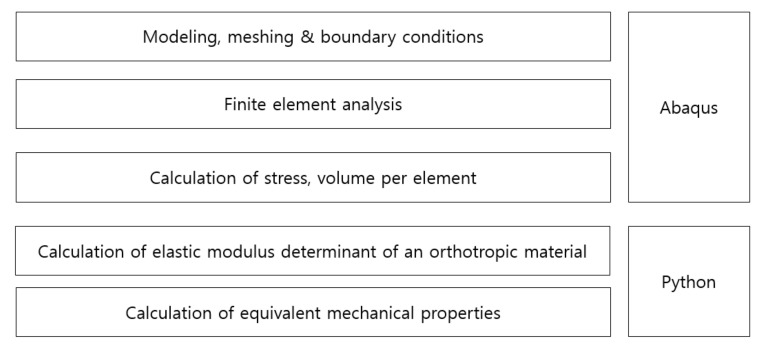
The procedure of calculation of equivalent mechanical properties.

The unit model described in Section 3.2 was modeled, and the grid was constructed using a hexagonal grid (C3D8R). A displacement of 0.1% relative to the length was applied as a tensile load and a shear load. The equivalent stress was calculated according to the process in Section 3.2, and the elastic modulus matrix was calculated using the equivalent strain. The procedure of this method is shown in Figure 9.

**Figure 10:**
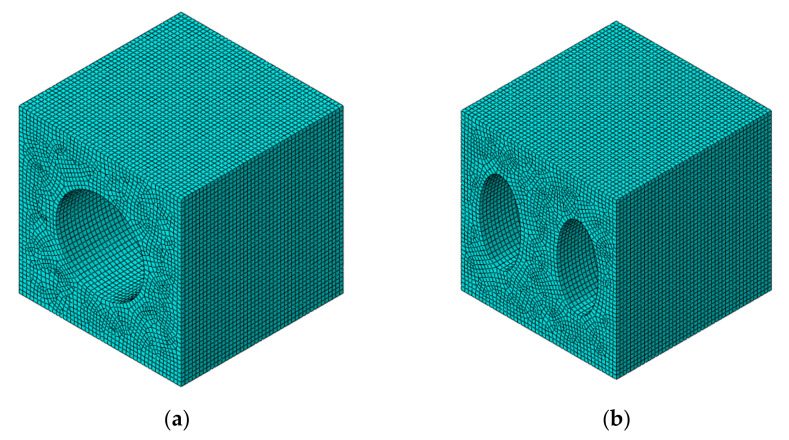
The shape of unit cell after meshing. (**a**) Circular pores. (**b**) Elliptical pores.

When simulating the circular pores, the length of cubic was 221.25 μm and the diameter of hole was 116.25 mm, which was based on real specimen. The size of mesh was 5 μm, and the shape of mesh was hexagonal grid with reduced integration (C3D8R), which is shown in Figure 10. Moreover, the number was mesh is over 76,000.

In case of elliptical pores, the length of cubic was 433.13 μm and the diameters of holes were 120.0 μm and 233.13 μm. The mesh style was as same as in the case of circular pores, but the size was 10μm and the number of mesh was over 77,000. It is shown in Figure 10.

**Figure 11:**
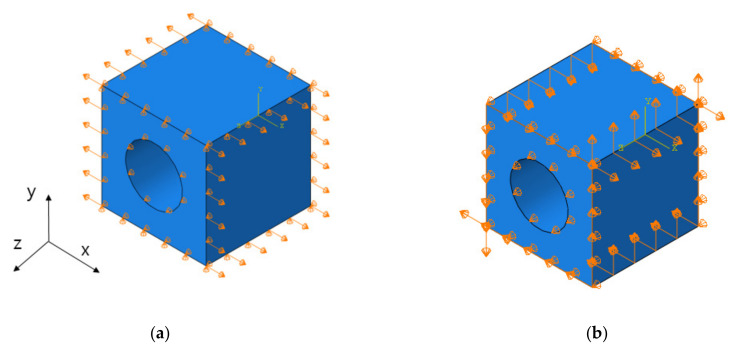
The boundary condition for strain. (**a**) X-direction tensile strain. (**b**) XY-direction shear strain.

**Figure 12:**
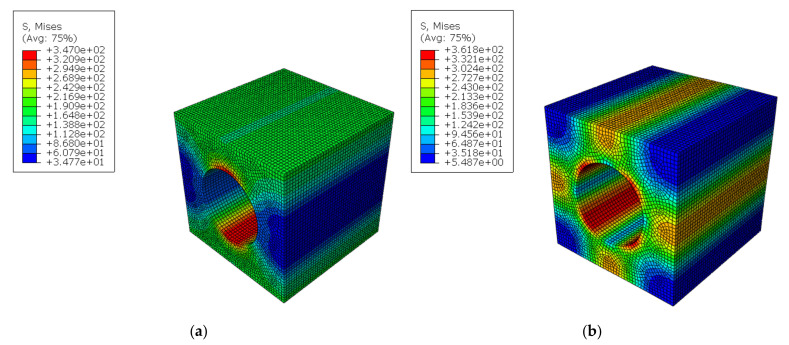
The distribution of stress with 0.1% strain condition. (**a**) X-direction tensile strain. (**b**) XY-direction shear scheme 11. S22, S33, S12, S13 and S23 (S11: tensile stress of X-direction, S22: tensile stress of Y-direction, S33: tensile stress of Z-direction, S12: shear stress of XY-direction, S13: shear stress of XZ-direction, S23: shear stress of YZ-direction) of each element were calculated and printed out. The calculation of weighted average concluded the equivalent stress of each direction at each step.

Tensile strain and shear strain were loaded with boundary condition, and 0.1% of strain was applied. The tensile strain, shear strain by each direction, was applied in each step. Step 1 was for tensile strain of X direction, step 2 was for tensile strain of Y direction, step 3 was for tensile strain of Z direction, step 4 was for shear strain of XY direction, step 5 was for shear strain of XZ direction, and step 6 was for shear strain of YZ direction. Examples are shown in Figure 11. Each step was independent from other steps, and each did not affect other steps. The results of FEA are shown in Figure 12.

The elastic modulus determinant of an orthotropic material (Equation (7)) can be solved with those stress data at each step. At this stage, the calculation for matrix such as inverse matrix and matrix product should be performed, Python with numpy library can solve this problem. With python, not only elastic modulus matrix but also the equivalent Young’s modulus and Poission’s ration were calculated

### 3.5. Simulation Results

Based on the calculation according to Section 3.2, the circular pores are shown in Table 3 and the elliptical pores are shown in Table 4.

## 4. Verification and Results through Experiments

### 4.1. Experimental Equipment and Methods

**Figure 13:**
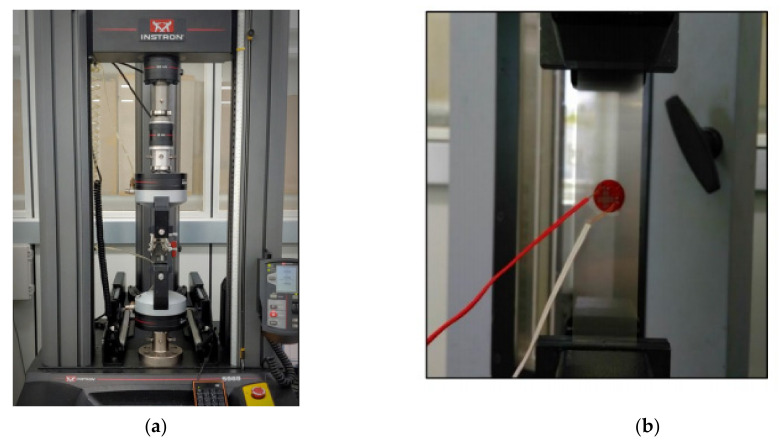
(**a**) UTM. (**b**) Two-axis strain gauge.

For the verification of this study, a test specimen was prepared and the elastic modulus and Poisson’s ratio were measured using a universal testing machine (UTM) (Instron, Norwood, MA, USA). As a universal testing machine, the Instron 5969 was used, and a two-axis strain gauge was used to measure the elastic modulus and Poisson’s ratio (Figure 13). Experiments and measurements were performed in compliance with KS M ISO 527-4, and the static material properties were measured at a tensile speed of 1 mm/min. For higher reliability, the experiment was performed six times and the average value was used.

### 4.2. Shape of Test Specimen

**Figure 14:**
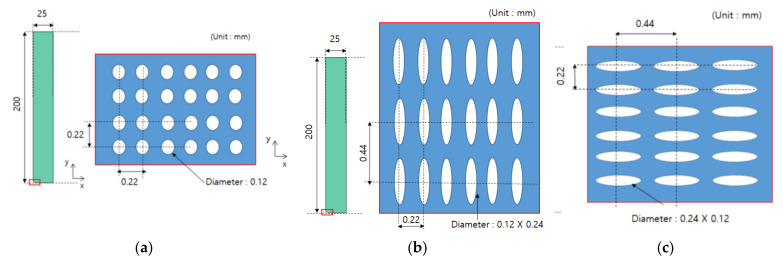
Types of test specimens. (**a**) Specimen with circular pores. (**b**) Specimen with elliptical pores—long axis in the tensile direction. (**c**) Specimen with elliptical pores—short axis in the tensile direction (Reprinted from Ref. [14]).

The test specimen was produced in a size of 25 mm × 200 mm × 0.03 mm, and an etching technique was used to prepare the specimen. STS304 was used as a material, and the experiment was performed with three types of specimens. Type I is a test specimen that simulates a circular pore, and Type II and Type III are test specimens that simulate elliptical pores. In each case, a specimen was prepared separately to measure the equivalent elastic modulus and the equivalent Poisson’s ratio. When measuring a tensile stress using UTM, it was measured in the Y direction. The shape of a specimen is summarized in Figure 14 [14].

### 4.3. Measurement Results

The results of six experiments with three types of specimens are shown in Table 5, Table 6 and Table 7. It was found that the range and standard deviation of the results of six experiments were not significant.

### 4.4. Comparison and Verification

Table 8, Table 9 and Table 10 compare the equivalent properties estimated from this study with those obtained through actual measurements.

**Figure 15:**
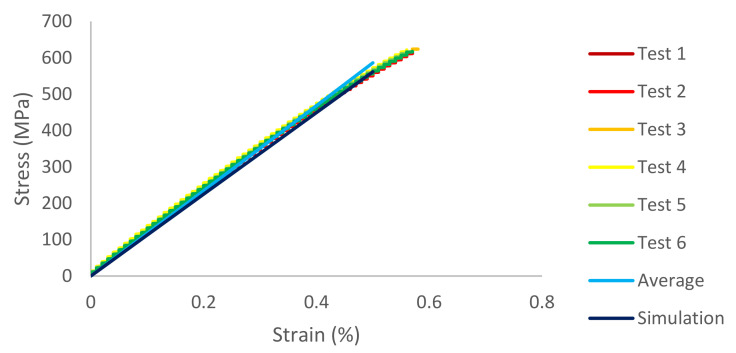
Stress–strain curve for circular pores (Type I).

**Figure 16:**
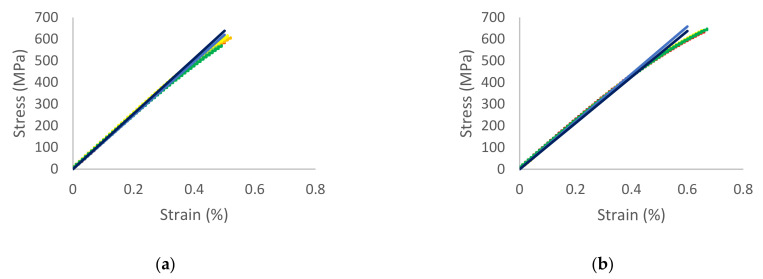
Stress–strain curve for elliptical pores. (**a**) Type II. (**b**) Type III.

In addition, the stress–strain curves for Type I, Type II, and Type III are shown in Figure 15 and Figure 16. As the standard deviation of each case is not large, the difference among tests and average is very small. Moreover, the difference between average and simulation is not big; the curves are similar.

## 5. Discussion

The purpose of this study is to estimate the elastic modulus and Poisson’s ratio of a material with irregular pores. When the size and arrangement of pores are irregular, the material properties should be analyzed using a completely anisotropic material. Therefore, more in-depth research is required. In this study, a research method that is feasible in the industrial field was proposed by estimating material properties more easily through working from the assumption that pores have the characteristics of 2D orthogonal anisotropy. To avoid the modeling and analysis of all pores, calculation for equivalent properties was performed using the representative volume element method. A Python program was developed to calculate equivalent properties based on the assumption that the pores are orthogonal anisotropic materials. To verify the results produced by the program, a specimen was produced by simulating circular pores and elliptical pores with horizontal/vertical asymmetry, and the simulation and measurement results were compared after measuring the specimen using UTM. We checked and verified three cases with our method. As differences between simulation and measurement are under 6.4%, our method is reliable and accurate. This research can be applied to many industries such as medicine and dentistry.

**Figure 17:**
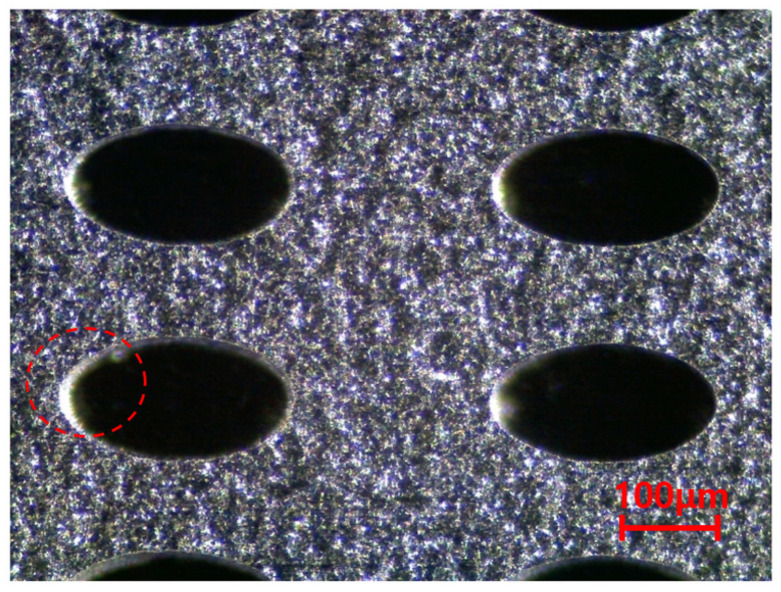
The shape of imperfect fabrication.

In the case of a specimen simulating pores in an elliptical shape, it was found that its production was difficult due to the shape. Thus, the fabrication was not perfect; as shown in Figure 17, the dimensions of top side and bottom side of ellipse were not same. As the shape of upper and lower sides was different, it might affect the results.

Because the shape and arrangement of pores are not uniform, the assumption of an orthotropic material has limitations when it comes to simulating the actual behavior. However, in the case of anisotropic elasticity, it is only possible to estimate the elastic modulus matrix through measurement, and it is difficult to say that the estimated elastic modulus matrix perfectly simulates a porous material. Although it does rely on some assumptions, the results of this study will be of great help in industrial design if they are applied to the finite element method using porous materials in the industrial field. Especially, this method can be useful for analysis of materials in medicine and dentistry, such as bacterial biofilm [21], bones, and porous tantalum. Research considering porosity is very important, as porosity of certain materials can influence the retention and formation of bacterial biofilm

## 6. Conclusions

In this study, the equivalent properties were calculated and verified by assuming that the material with pores was an orthotropic material while applying the representative volume element method.

1. In the case of a finite element method using porous or composite material, it is inefficient to perform the analysis using material modeling. The equivalent properties of a material were estimated by applying the representative volume element method.
2. Working from the assumption that the pores are horizontally/vertically asymmetrical, an elastic modulus matrix of an orthogonal anisotropic material was constructed. The equivalent elastic modulus and equivalent Poisson’s ratio of a representative volumetric element were calculated using the equivalent strain and equivalent stress.
3. Based on the element volume and element stress values derived from the finite element method program, the representative stress value and elastic modulus matrix were calculated using Python. In addition, the equivalent material properties were derived using the calculated elastic modulus matrix.
4. A thin-plate specimen made of STS304 was etched in a specific pattern to simulate pores. The elastic modulus and Poisson’s ratio were measured using UTM and verified through comparison with simulation results.
5. This research can be applied to many industries such as medicine and dentistry, which treat porous materials such as bacterial biofilm, bones, teeth, and porous tantalum. As porosity of certain materials can influence the retention and formation of bacterial biofilm, this research is very powerful for analysis for materials with porosity.

## Figures and Tables

**Table 1:** Comparison of design proposal and actual results (unit: μm).

			Design	Real
Circular pore	Length of unit cell		220.0	221.25
	Hole diameter		120.0	116.25
Elliptical pore	Length of unit cell		440.0	433.13
	Hole diameter	Short	120.0	120.00
		Long	240.0	233.13

**Table 2:** Mechanical properties of SUS304 [13,14].

Contents	Value	Unit
Density	8000	kg/m^3^
Modulus of elasticity	193.0	GPa
Poisson’s ratio	0.29	-

**Table 3:** Equivalent properties with simulation for circular pores.

Contents	Value	Unit
Elastic modulus (*E*_x_)	112.3	GPa
Elastic modulus (*E*_y_)	112.3	GPa
Elastic modulus (*E*_z_)	147.9	GPa
Poisson’s ratio (*ν*_yx_)	0.230	m/m
Poisson’s ratio (*ν*_xy_)	0.230	m/m
Poisson’s ratio (*ν*_zx_)	0.290	m/m
Poisson’s ratio (*ν*_zy_)	0.290	m/m

**Table 4:** Equivalent properties with simulation for elliptical pores.

Contents	Value	Unit
Elastic modulus (*E*_x_)	106.2	GPa
Elastic modulus (*E*_y_)	127.6	GPa
Elastic modulus (*E*_z_)	150.5	GPa
Poisson’s ratio (*ν*_yx_)	0.235	m/m
Poisson’s ratio (*ν*_xy_)	0.196	m/m
Poisson’s ratio (*ν*_zx_)	0.290	m/m
Poisson’s ratio (*ν*_zy_)	0.290	m/m

**Table 5:** Equivalent properties of Specimen Type I [13].

Test No.	Modulus of Elasticity (GPa)	Poisson’s Ratio (mm/mm)
1	116.0	0.226
2	117.0	0.228
3	117.0	0.243
4	118.0	0.248
5	118.0	0.242
6	117.0	0.231
Average	117.17	0.2363
Standard Deviation	0.687	0.0083

**Table 6:** Equivalent properties of Specimen Type II [14].

Test No.	Modulus of Elasticity (GPa)	Poisson’s Ratio (mm/mm)
1	124.0	0.211
2	124.0	0.233
3	124.0	0.212
4	127.0	0.223
5	123.0	0.219
6	122.0	0.226
Average	124.0	0.221
Standard Deviation	1.53	0.0077

**Table 7:** Equivalent properties of Specimen Type III [14].

Test No.	Modulus of Elasticity (GPa)	Poisson’s Ratio (mm/mm)
1	110.0	0.196
2	109.0	0.196
3	111.0	0.188
4	110.0	0.206
5	109.0	0.194
6	109.0	0.199
Average	109.7	0.197
Standard Deviation	0.75	0.0054

**Table 8:** Difference between simulation and measurement for Type I.

	Modulus of Elasticity (GPa)	Poisson’s Ratio (mm/mm)
Simulation	112.3	0.232
Measurement	117.2	0.236
Difference (%)	4.18 (%)	1.69 (%)

**Table 9:** Difference between simulation and measurement for Type II.

	Modulus of Elasticity (*E_y_*, GPa)	Poisson’s Ratio (*ν_yx_*, m/m)
Simulation	127.6	0.236
Measurement	124.0	0.221
Difference (%)	2.82 (%)	6.36 (%)

**Table 10:** Difference between simulation and measurement for Type III.

	Modulus of Elasticity (*E_x_*_,_ GPa)	Poisson’s Ratio (*ν_xy_*, m/m)
Simulation	106.2	0.196
Measurement	109.7	0.197
Difference (%)	3.19 (%)	0.51 (%)

## Data Availability

The data presented in this study are available on request from the corresponding author.

## References

[1] Katsoulidis A.P., Antypov D., Whitehead G.F.S., Carrington E.J., Adams D.J., Berry N.G., Darling G.R., Dyer M.S., Rosseinsky M.J. (2019). Chemical control of structure and guest uptake by a conformationally mobile porous material. Nature.

[2] Slater A.G., Cooper A.I. (2015). Porous materials function-led design of new porous materials. Science.

[3] Nomura T., Okinaka N., Akiyama T. (2009). Impregnation of porous material with phase change material for thermal energy storage. Mater. Chem. Phys..

[4] Trunh L., Lee D. (2020). Welding of Thin Tab and Battery Case for Lithium-ion Battery Cylindrical Cell Using Nanosecond Pulsed Fiber Laser. J. Weld. Jt..

[5] Mour M., Das D., Winkler T., Hoenig E., Mielke G., Morlock M.M., Schilling A.F. (2010). Advances in Porous Biomaterials for Dental and Orthopaedic Applications. Materials.

[6] Liu Y., Bao C., Wismeijer D., Wu G. (2015). The physicochemical/biological properties of porous tantalum and the potential surface modification techniques to improve its clinical application in dental implantology. Mater. Sci. Eng. C.

[7] Wang Z., Chang K., Muzaffer S. (2020). Fatigue Analysis of the Effects of Incomplete Penetration Defects on Fatigue Crack Initiation Points in Butt-Welded Members. J. Weld. Jt..

[8] Wu C., Kim J. (2020). Review on Mitigation of Welding-Induced Distortion Based on FEM Analysis. J. Weld. Jt..

[9] Babu K.P., Mohite P.M., Upadhyah C.S. (2018). Development of an RVE and its stiffness predictions based on mathematical homogenization theory for short fibre composites. Int. J. Solids Struct..

[10] Breuer K., Stommel M. (2020). RVE modelling of short fiber reinforced thermoplastics with discrete fiber orientation and fiber length distribution. SN Appl. Sci..

[11] Bargmann S., Klusemann B., Markmann J., Schnabel J.E., Schneider K., Soyarslan C., Wilmers J. (2018). Generation of 3D representative volume elements for heterogeneous materials: A review. Prog. Mater. Sci..

[12] Pan Y., Iorga L., Pelegri A.A. (2008). Generation of 3D representative volume elements for heterogeneous materials: A review. Compsites Sci. Technol..

[13] Pyo C. (2021). A Study for Estimation of Equivalent Mechanical Properties of Materials with Porosity Part I. Isotropic Elasticity in Plane Stress. Korean Soc. Mech. Technol..

[14] Pyo C. (2021). A Study for Estimation of Equivalent Mechanical Properties of Materials with Porosity Part II. Orthotropic Elasticity in Plane Stress. Korean Soc. Mech. Technol..

[15] Schmitz A., Horst P. (2014). A finite element unit-cell method for homogenized mechanical properties of heterogeneous plates. Compos. Part A Appl. Sci. Manuf..

[16] Zheng Q., Fan H. (2021). Equivalent continuum method of plane-stress dominated plate-lattice materials. Thin-Walled Struct..

[17] Daniel I.M., Ishai O. (2006). Engineering Mechanics of Composite Materials.

[18] Pyo C., Kim J., Kim J. (2020). Estimation of Heat Source Model’s Parameters for GMAW with Non-linear Global Optimization—Part I: Application of Multi-island Genetic Algorithm. Metals.

[19] Deng D., Murakawa H. (2006). Numerical simulation of temperature field and residual stress in multi-pass welds in stainless steel pipe and comparison with experimental measurements. Comput. Mater. Sci..

[20] Sixun T., Haipeng G., Hao L., Lei Q. The FEM Model Driven Method of the Static Performance Calculation for Gas Joumal Bearing by Python. Proceedings of the 2018 IEEE International Conference on Mechatronics and Automation.

[21] Stafslien S., Bahr J., Feser J., Weisz J., Chisholm B., Ready T., Boudjou P. (2006). Combinatorial Materials Research Applied to the Development of New Surface Coatings I: A Multiwell Plate Screening Method for the High-Throughput Assessment of Bacterial Biofilm Retention on Surfaces. J. Comb. Chem..

